# *Mycoplasma pneumoniae* pneumonia associated thrombosis at Beijing Children’s hospital

**DOI:** 10.1186/s12879-020-4774-9

**Published:** 2020-01-16

**Authors:** Jinrong Liu, Ruxuan He, Runhui Wu, Bei Wang, Hui Xu, Yue Zhang, Huimin Li, Shunying Zhao

**Affiliations:** 1 Department of Respiratory Medicine, Beijing Children’s Hospital, National Centre for Children’s Health, Capital Medical University, NO.56, Nanlishi Road, Xicheng District, Beijing, 100045 People’s Republic of China; 2 Department of Pediatrics, Peking University First Hospital, Beijing, 100034 China; 3 Hematology Oncology Center, Beijing Children’s Hospital, National Centre for Children’s Health, Capital Medical University, Beijing, 100045 China; 4 Department of Radiology, Beijing Children’s Hospital, National Centre for Children’s Health, Capital Medical University, Beijing, 100045 China

**Keywords:** Severe, Mycoplasma pneumoniae, Pneumonia, Thrombosis, Children

## Abstract

**Background:**

With the increase of awareness of *mycoplasma pneumoniae* pneumonia (MPP), we found thrombosis in severe MPP (SMPP) was not rare. The aim of the study was to investigate the clinical characteristics, treatment, and long-term prognosis of MPP-associated thrombosis.

**Methods:**

We retrospectively reviewed the medical records of 43 children with MPP-associated thrombosis between January 2013 and June 2019 at Beijing Children’s Hospital. The results of blood coagulation studies, autoimmune antibody, thrombophilia screening, contrast-enhanced lung computed tomography, echocardiography, and blood vessel ultrasonography were analyzed, as were treatment outcomes.

**Results:**

Forty-two patients were diagnosed with SMPP. D-dimer was higher than 5.0 mg/L in 58.1% (25/43) of patients. The mean D-dimer level was 11.1 ± 12.4 mg/L. Anticardiolipin-IgM was positive in 60.0% of patients, β2-glycoprotein-IgM in 64.0%, and lupus anticoagulant in 42.1%. Chest imaging revealed pulmonary consolidation with lobe distribution in all patients (2/3–1 lobe in 10 patients, > 1 lobe in 29 patients). In our experience, thrombosis can occur in a vessel of any part of the body, and it can be initially detected as late as 31 days after disease onset. Thrombosis in the brain and abdomen can occur early, at 5 days after disease onset. Pulmonary vessels were the most commonly involved sites in the current study, and accordingly chest pain was the most common symptom (32.6%), followed by neurological symptoms (14.0%) and abdominal pain (9.3%). Thirty-five percent of patients were asymptomatic with regard to thrombosis. All patients underwent anticoagulant therapy, and thrombus absorption took > 3 months in most patients. All patients were followed until October 2019, at which time 41 were asymptomatic and 2 had mild recurrent cough.

**Conclusions:**

SMPP with pulmonary consolidation (> 2/3 lobe) was the most strongly associated risk factor for thrombosis. Thrombosis-associated symptoms may be subtle, even absent. Elevated D-dimer, specifically > 11.1 mg/L (even > 5.0 mg/L), would assist in the early diagnosis of thrombosis. The long-term prognosis of thrombosis was good after timely administration of anticoagulant therapy.

## Background

*Mycoplasma pneumoniae* (MP) pneumonia (MPP) accounts for approximately 10 to 40% of community-acquired pneumonia (CAP) cases in children [1–3], which is traditionally described as mild and self-limited; however, in the near 10 years, many refractory, severe, fulminant or even fatal cases of MPP have been reported particularly in Eastern Asia [4–7]. In addition, MPP can lead to some complications such as necrotizing pneumonia (NP) [8], airway obliterans (AO) [4, 7, 9], hemolytic anemia [10], and thrombosis [11, 12].

Vascular complication is one of the rarest extrapulmonary complications. It has been reported that children with MPP had a high risk of blood coagulation and thrombosis [11, 13, 14]. However, there have been only a few reported cases of MPP-associated thrombosis, and data on its risk factors, clinical characteristics, and long-term prognosis of the larger studies are scarce. With the increase of awareness of this disease, we found thrombosis in severe MPP (SMPP) was not rare. Here, we describe 43 pediatric MPP-associated thrombosis cases with diverse clinical manifestations. To our knowledge, the sample size of this study is the largest, compared with the previous publications.

## Methods

### Study population

This study was conducted between January 2013 and June 2019 at Beijing Children’s Hospital affiliated to Capital Medical University, National Center for Children’s Health, the largest children’s hospital in China. All children hospitalized with MPP-associated thrombosis at Department of Respiratory Medicine were enrolled in this study. The study was approved by the Ethics Committee of Beijing Children’s Hospital, and informed written consent was obtained from guardian of all the patients at the beginning of admission to our department.

The medical records of all subjects were retrospectively reviewed. Information including clinical presentations, the levels of inflammatory markers such as C-reactive protein (CRP) and lactate dehydrogenase (LDH), and the results of blood coagulation studies and thrombophilia screen such as anticardiolipin (aCL) antibodies and lupus anticoagulant (LA) were all collected. In addition, the findings of bronchoscopy, contrast-enhanced lung CT, echocardiography, and blood vessel ultrasonography, treatment outcomes were all recorded.

### Diagnostic criteria

MPP was diagnosed based on the followings [15]: (1) clinical presentation (fever, cough); (2) chest imaging with infiltrates; (3) serum anti-MP IgM titer ≥1:320 or four-fold rising titer in acute and convalescent serum specimens. In this study, SMPP defined as MPP with one of the followings [15] except (5): (1) poor general condition; (2) increased respiratory rate (> 50/min); (3) dyspnoea and cyanosis; (4) multilobe involvement or ⩾2/3 lung involvement; (5) extrapulmonary complication; (6) pleural effusion and (7) pulse oxygen saturation in room air ⩽92%.

### Statistical analyses

SPSS-Version 24 (IBM Corp., Armonk, NY, US) was utilized for statistical analysis of the collected data. All value data presented are expressed as mean ± standard deviation (SD).

## Results

### Study population

**Fig. 1:**
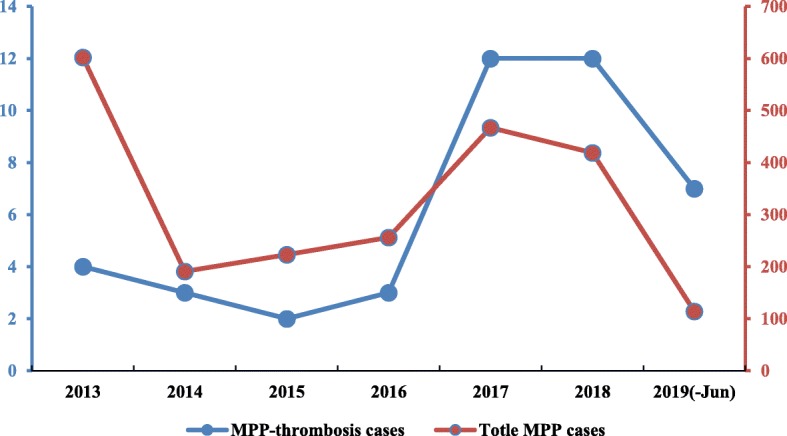
The annual case number of MPP-associated thrombosis between January 2013 and June 2019

A total 43 patients (from 4-years-1-month to 14-years-2-months, mean age 7-years-11-months) were finally enrolled. As shown in Fig. 1, the number of patients with thrombosis enrolled in the recent 3 years was the most. 53.5% (*n* = 23) were male, and 46.5% (*n* = 20) were female. Past history revealed allergic purpura in 1 patient. All patients were treated for at least 3 days at other hospitals or our outpatient clinic before admission. Forty-two patients were diagnosed with SMPP.

**Fig. 2:**
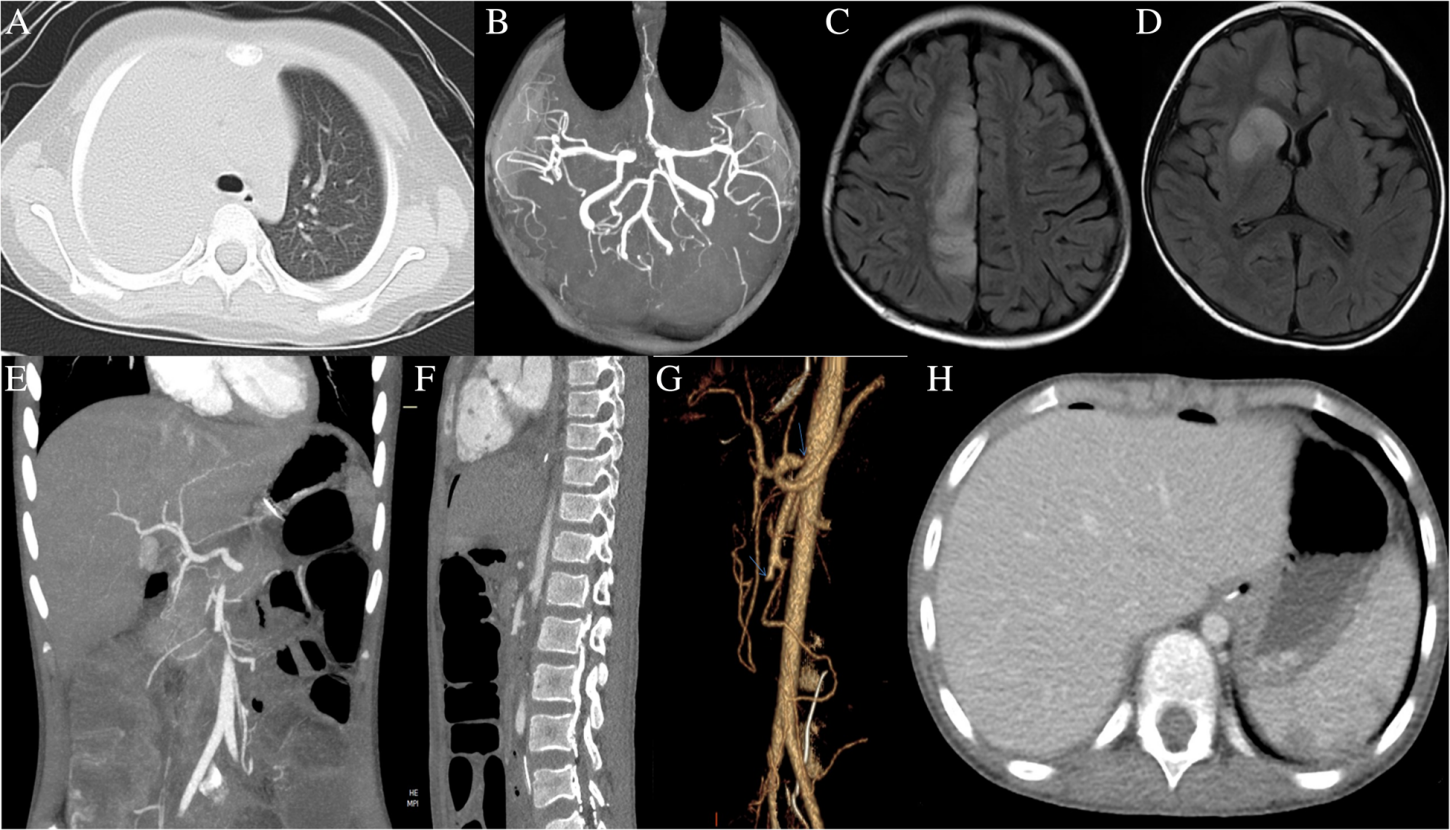
Chest imaging revealed consolidation with high density in the right upper lung (**a**). 3D-TOF MRA image of the brain didn’t reveal the A1 segment of the right anterior cerebral artery (**b**), and the T2 FLAIR transverse image showed a patchy high signal in the right parietal cerebral palsy and the right basal ganglia (**c**, **d**). Abdominal enhanced CT and 3D vascular reconstruction revealed the superior mesenteric artery thrombosis (**e**, **f**, **g**), and multiple small infarction in the spleen (**e**, **h**)

A family history of stroke (the grandparents) was identified in 4 patients. Among them, whole exome sequencing (WES) was performed in three families. WES identified a heterozygous variant c.2346G > A in *MTHFR* in Case 1, c.1342C > G in *MTHFR* in Case 2, and c.1334C > T in *DSG2* in Case 3 which was found to be inherited from the patient’s heterozygous father or mother. MP polymerase chain reaction (PCR) was performed in the bronchoalveolar lavage fluid (BALF) of 9 patients and in the peripheral blood of 3 patients at the early stage. All BALF samples and only 1 blood sample (Case 1) were MP-PCR-positive. Case 1 accompanied with type I respiratory failure, pulmonary artery (PA) and pulmonary vein (PV) thrombosis. In Case 2 there was superficial vein of the left cubital fossa and right subsegmental PA thrombosis. In Case 3 there was left cephalic vein thrombotic superficial phlebitis, PA, and right ventricular thrombosis (13.3 × 7.4 mm) attached to tricuspid valve chordae tendineae. Additionally, one patient (Case 4) underwent partial bowel resection because of intestinal obstruction and enteral necrosis due to superior mesenteric artery and celiac trunk artery thrombosis (Fig. 2e-g). That patient also had spleen infarction (Fig. 2h). In one patient (Case 5) thrombosis was accompanied by cerebral infarction and pancreatitis.

### Clinical characteristics of patients

The average duration of disease before hospitalization to our department was 20.9 ± 17.9 days [from 7 days to 40 days in 40 patients, 60 days in 2 patients and 6 months in 1 patient (these 3 patients had recurrent fever and cough after acute MPP stage)]. The patients presented with persistent high fever and cough (*n* = 43, 100%), type I respiratory failure (*n* = 8; 18.6%), chest pain (*n* = 14; 32.6%), and hemoptysis (*n* = 2; 4.7%) (Table 1). Other clinical symptoms are shown in Table 1. The long disease duration and high rate of respiratory failure suggested the severity of SMPP.

**Table 1:** The clinical symptoms and relevant involved vessels of pediatric MPP-associated thrombosis

Clinical symptoms and involved vessels	Case (n)	Percentage	Onset time (days)
Chest pain	14	32.6% (14/43)	7–31
Pulmonary artery (including cases 1–3, 6–9)	11		
Pulmonary vein (including case 1)	3		
Hemoptysis	2	4.7% (2/43)	12–20
Pulmonary artery, and necrotizing pneumonia	2		
Neurological symptoms	6	14.0% (6/43)	5–9
Cerebral infarction and hemiplegia such as disorder of consciousness, weakness of unilateral limb, dysphagia etc.	3		
Middle cerebral artery	2		
Anterior cerebral artery (case 10)	1		
Disorder of consciousness and convulsion	2		
Sigmoid sinus	1		
Craniocerebral vein (case 6)	1		
Headache, dizziness	1		
Unknown (case 8)	1		
Abdominal pain [including a case of pancreatitis (case 5) or intestinal obstruction]	4	9.3% (4/43)	5–30
Pulmonary artery singly	2		
Splenic artery (cases 4 and 8)	2		
Celiac trunk artery (case 4)	1		
Superior mesenteric artery (case 4)	1		
Chest tightness	1	2.3% (1/43)	10
Pulmonary vein and left atrium	1		
Shoulder pain (one side)	1	2.3% (1/43)	33
Superficial vein of cubital fossa (case 2)	1		
Back pain (one side)	1	2.3% (1/43)	30
Pulmonary vein	1		
Swelling of lower limb (one side)	3	7.0% (3/43)	9–15
Common iliac vein (case 9)	1		
Common femoral vein (including case 9)	2		
Great saphenous vein	1		
Low temperature of limb skin (one side)	1	2.3% (1/43)	9
Popliteal artery and posterior tibial artery	1		
Asymptomatic about thrombosis	15	34.9% (15/43)	7–18

### Laboratory data

#### Inflammatory markers especially CRP and LDH significantly increased

The mean peripheral white blood cells (WBC) counts, neutrophil (N) percentage, CRP and LDH at the early stage are shown in Table 2. CRP and LDH decreased to be normal within 1 month after disease onset. The platelet (PLT) counts were normal before 15 days of disease in all patients. Then PLT counts gradually increased over time in most patients and finally PLT counts were even high up to 982 × 10^9^/L within 2 months.

**Table 2:** Laboratory data of pediatric MPP-associated thrombosis

WBC (×10^9^/L)	N (%)	CRP (mg/L)	LDH (IU/L)	Elevated ALT (IU/L)	FIB (g/L)	D-dimer (mg/L)
11.6 ± 4.2	77.6 ± 11.7	97.5 ± 87.3	735.1 ± 641.9	228.0 ± 419.6	4.5 ± 2.2	11.1 ± 12.4

*WBC* white blood cells, *N* neutrophil, *CRP* C-reactive protein, *LDH* lactate dehydrogenase, *ALT* alanine aminotransferase, *FIB* fibrinogen. Normal range: CRP < 8 mg/L; LDH < 240 IU/L; ALT< 40 IU/L; FIB:2-4 g/L; D-dimer< 0.243 mg/L

#### Elevated liver enzymes were found in 60.5% of patients without obvious evidence of Epstein-Barr Virus (EBV) infection

Elevated alanine aminotransferase (ALT) was detected in 60.5% (26/43) of patients (Table 2). Among these patients, serum EBV-CA-IgM and/or EBV-EA-IgA was positive in 5, and EBV-DNA was positive in 4 (10^2^–10^3^ copies). None of patients had any other evidence of EBV infection, and none were treated with anti-EBV drugs.

#### Blood coagulation studies especially D-dimer revealed a hypercoagulable state

The peak level of fibrinogen (FIB) and D-dimer was detected within 6–15 days after disease and their concentrations were 4.5 ± 2.2 g/L and 11.1 ± 12.4 mg/L, respectively (Table 2). D-dimer was higher than 5.0 mg/L in 58.1% (25/43) of patients and higher than 2.0 mg/L in 93.0% (40/43) of patients.

#### Autoimmune antibodies revealed positive in most patients

Anti-nuclear antibodies (ANAs) were positive in 51.2% (22/43) of patients, and titers ranged from 1:10 to 1:640. Anti-ENA-antibodies (mainly anti-Sm) were positive in 16.3% (7/43) of patients. Antineutrophil cytoplasmic antibody (pANCA and cANCA) was positive in 1/43 (2.3%) patients (Table 3). ANAs were still positive in 3 patients after 6 months, but those patients did not have any symptoms of autoimmune disease.

**Table 3:** Positive antibodies in pediatric MPP-associated thrombosis

ANAs	Anti-ENA-antibodies	aCL-IgM	β2-glycoprotein-IgM	LA
51.2%	16.3%	60.0%	64.0%	42.1%

*ANAs* anti-nuclear antibodies, *aCL* anti-nuclear antibodies, *LA* lupus anticoagulant

#### Thrombophilia screening mainly aCL antibodies revealed positive in most patients

aCL-IgM was positive in 60% (15/25) of patients, β2-glycoprotein-IgM was positive in 64% (16/25), and LA was positive in 42.1% (8/19) of patients. Protein S activity was low in 5.1% (2/39) of patients (Table 3). Antithrombin-III and serum homocysteine levels were normal in all patients, as was protein C activity. Six months after initial disease onset the aforementioned antibodies that had been positive were negative, and protein S activity was normal.

#### Chest imaging revealed pulmonary consolidation in all patients, subsequently 44.2% of patients had necrotizing pneumonia (NP)

**Fig. 3:**
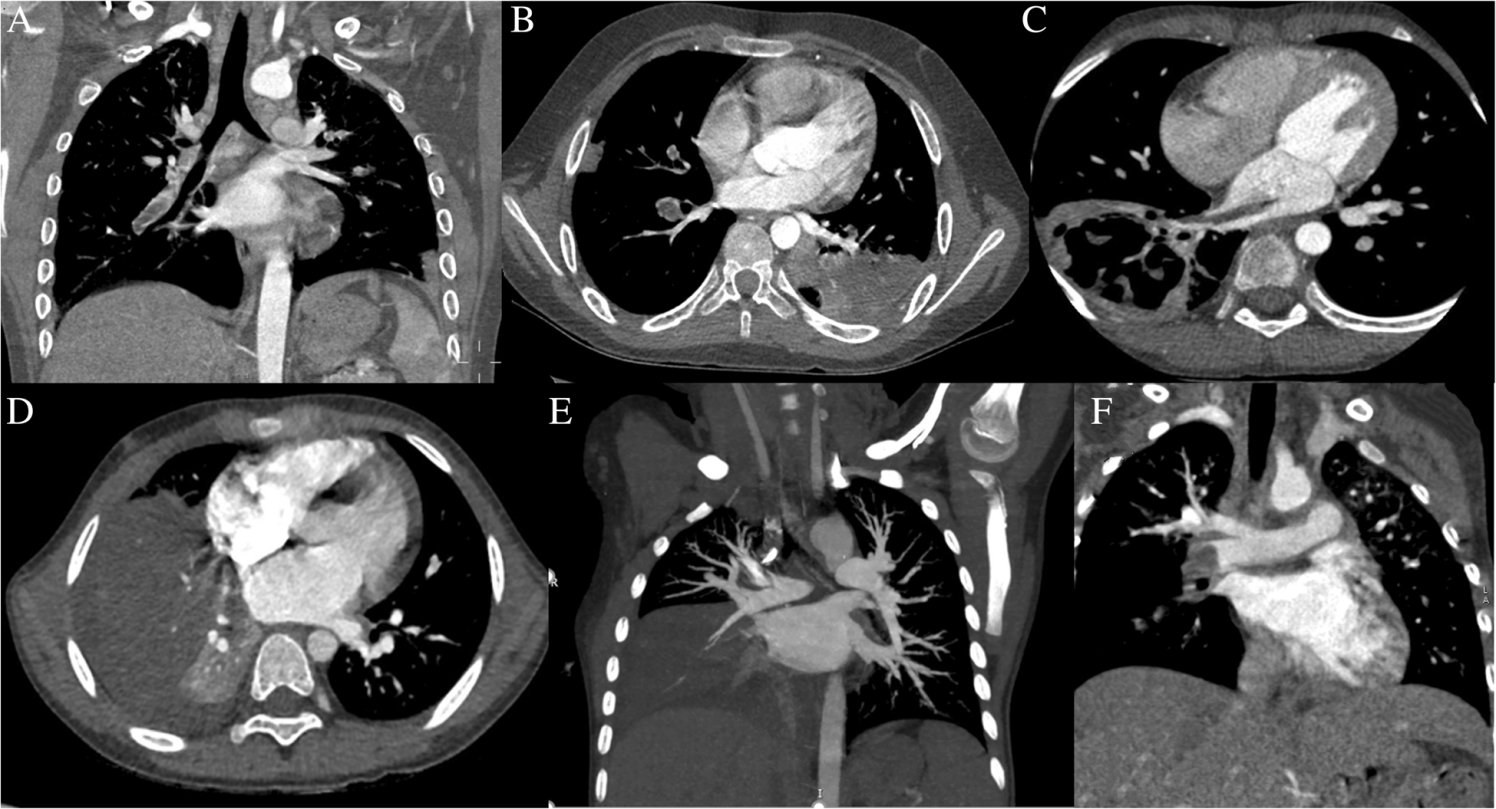
Lung enhanced CT and 3D vascular reconstruction revealed right lower pulmonary artery thrombosis (**a**, **b**), necrosis within consolidation of the left lower lung (**b**), low-density necrosis of the right lower lung with cavity formation, and the strip filling defect in the right lower pulmonary vein extended to the left atrium (**c**), necrosis of the right lower lung (**d**) with right lower pulmonary vein occlusion (**d**, **e**), and right pulmonary artery thrombosis (**f**)

At the early stage of disease, chest imaging revealed pulmonary consolidation with lobe distribution (1/3–2/3 pulmonary lobe in 4 patients, 2/3–1 lobe in 10 patients; ≥1 lobe in 29 patients) in 100% (43/43) of patients and pleural effusion in 74.4% (32/43) of patients (Fig. 2a). NP occurred in 44.2% (19/43) of patients between 13 and 58 days of disease (Fig. 3c, d, Table 4).

**Table 4:** Findings of chest imaging and bronchoscopy in pediatric MPP-associated thrombosis

Chest imaging (n/%)			Bronchoscopy (n/%)			
Early stage		Late stage	Early stage		Late stage	
Consolidation	Pleural effusion	NP	Mucus plug	Mucous necrosis	Airway stenosis	AO
100 (100%)	32 (74.4%)	19 (44.2%)	30 (73.2%)	12 (29.3%)	4 (9.8%)	9 (22.0%)

*NP* necrotizing pneumonia, *AO* airway obliterans

#### Bronchoscopy revealed airway hypersecretion at the early stage and airway deformation in the late stage

Bronchoscopy was performed in 41 patients. At the early stage, bronchoscopy revealed viscous secretion in 100% (41/41) of patients, mucus plugs in 73.2% (30/41) of patients (including 4 patients with plastic bronchitis), and mucous necrosis in 29.3% (12/41) of patients. In the late stage, 9.8% (4/41) of patients had airway stenosis and 22.0% (9/41) of patients had AO (Table 4).

#### Thrombus sites

As shown in Table 5, the PA was the most commonly involved vessel (Fig. 3a, b, f). In 21 patients only the PA was involved. Of these 21 patients, PA thrombosis occurred on the same side as the pulmonary consolidation in 10. In 5 patients only the PV was involved. In 1 patient (Case 6) PA, craniocerebral vein, and internal jugular vein thrombosis were all detected. In 1 patient (Case 7) local thickening in the media of the right subclavian artery was detected, suggesting arteritis (Tables 1 and 5).Magnetic resonance imaging (MRI), magnetic resonance artery (MRA) imaging, and magnetic resonance vein (MRV) imaging of the brain were performed in 10 patients, and revealed thrombosis in 5 patients (including Case 6, Tables 1 and 5, Fig. 2b-d). Brain MRI revealed ischemic foci (patchy abnormal signals) in the white matter of bilateral frontal, parietal, and occipital lobes in 1 patient (Case 8), suggesting thrombosis of multiple small arteries, because the patient had multi-thrombosis in other parts of the body (Tables 1 and 5).

**Table 5:** The involved vessels in patients with pediatric MPP-associated thrombosis

Site	Case (n)	Percentage
Brain	6	14.0% (6/43)
Middle cerebral artery	2	
Anterior cerebral artery (case 10)	1	
Craniocerebral vein (case 6)	1	
Sigmoid sinus	1	
Unknown small artery (case 8)	1	
Neck	1	2.3% (1/43)
Internal jugular vein (case 6)	1	
Lung	35	81.4% (35/43)
Pulmonary artery (including cases1–3,6–9)	30	
Pulmonary vein (including case 1)	8	
Heart	4	9.3% (4/43)
Tricuspid valve chordae tendineae (including case 3)	2	
Under the tricuspid valve	1	
Left atrium	1	
Abdomen	2	4.7% (2/43)
Splenic artery (cases 4 and 8)	2	
Celiac trunk artery (case 4)	1	
Superior mesenteric artery (case 4)	1	
Upper limbs	3	7.0% (3/43)
Cephalic vein (cases 3 and 8)	2	
Superficial vein of cubital fossa (case 2)	1	
Lower limbs	4	9.3% (4/43)
Popliteal artery	1	
Posterior tibial artery	1	
External iliac vein	2	
Common iliac vein (case 9)	1	
Common femoral vein (including case 9)	2	
Great saphenous vein	1	
Internal iliac vein	1	

#### Treatment and clinical outcomes

All patients were treated with azithromycin because of its low minimum inhibitory concentration against MP and anti-inflammatory effect. Moxifloxacin was simultaneously administered to 15 patients. Minocycline was administered to 2 patients after azithromycin was discontinued. Long-term anticoagulant therapy, mainly low molecular weight heparin (LMWH) was administered to all patients. Two patients with NP accompanied with PA thrombosis had slight hemoptysis, and one patient was allergic to LMWH so it had to be withdrawn after 1 month. Methylprednisolone (2–30 mg/kg.d) was administered to 41 patients. High-dose methylprednisolone (10–30 mg/kg.d) was administered to 10 patients, and warfarin was administered to 8 patients. Urokinase thrombolytic therapy was administered to 1 patient (Case 9) (Tables 1 and 5). Mild PA hypertension (PAH) and tricuspid regurgitation 2 months after disease onset were present in Case 9, but the PAH was no longer present 6 months after disease onset.

All patients were followed until October 2019, at which time 41 patients were asymptomatic and 2 had mild recurrent cough. After the initiation of anticoagulant therapy, thrombus absorption took > 3 months in most patients and 1.5–3 months in few patients, but the thrombosis-associated symptoms disappeared within 1 month in most patients. Notably, it can take up to 12 months for lower extremity deep vein thrombosis (DVT) to be absorbed. Intracranial venous occlusion (Case 6, age 8 years) and cerebral anterior artery occlusion (Case 10, age 5 years) each occurred in 1 patient. In 2 cases there was PV occlusion (including Case 1, Fig. 3d, e). Echocardiography depicted strong echo with punctate slight calcification on the tricuspid valve or tricuspid valve chordae tendineae in 2 patients.

## Discussion

Pediatric thrombosis is uncommon, the knowledge of which including pulmonary embolism (PE) remains fragmented. The most common precipitating factor is the presence of a central venous access device [16]. MPP is CAP occurring primarily in previously healthy school age children. In this study, 42 patients (97.7%) were diagnosed with SMPP, which was consistent with most previous case reports [17, 18]. Only a few cases were reported thrombosis was associated with isolated MP infection without MPP [19, 20]. This suggests that SMPP is the most strongly associated risk factor for MP-associated thrombosis. The severity of MPP was associated with fever duration, the levels of inflammatory markers such as CRP and LDH, and the severity of radiography findings such as lobar consolidation [21–24], which are strongly correlated with measures of the severity of airway damage such as mucous necrosis and subsequent airway remodeling [4]. The present study again confirmed the above associations, and the presence of an excessive systemic and local airway immune response in SMPP. MPP-associated hepatitis is not uncommon and has a relatively good prognosis [25]. It ranged from 7.7 to 30% [25], but we found a high incidence of 60.5%, which suggested the more serious MPP and more stronger autoimmune response in our patients. EBV-related testing revealed EBV infection in 5 patients. None of patients had any other evidence of EBV infection, and none were treated with anti-EBV drugs, which suggested the elevated liver enzymes were mainly associated with MP infection. Therefore, it is possible that EBV-IgM/IgA were cross-reactive with MP-IgM/IgA. In addition, EBV viremia suggested an EBV reactivation by MP infection. Early corticosteroid therapy was very important, and may prevent disease progression in MPP [26]. In the current study some patients did not receive corticosteroid treatment at the optimal time, which is during the early stage of MPP.

Biomarkers such as CRP and D-dimer levels were close related to severity of CAP [4, 22, 23, 27]. Plasma D-dimer is a degradation product of cross-linked fibrin and D-dimer values are elevated in the presence of acute clots [28]. In our study, D-dimer was 11.1 ± 12.4 mg/L and it was higher than 5.0 mg/L in 58.1% of patients, which suggested D-dimer > 5.0 mg/L, particularly > 11.1 mg/L would help the early diagnosis of thrombosis. D-dimer was normal or slightly elevated (< 2 mg/L) only in three patients, and the reason is perhaps that we did not timely monitor its peak. Higher D-dimer levels were related to significantly higher clot burden [29]. D-dimer was high up to 50.529 mg/L in Case 8 who had multiple thrombosis in PA, splenic artery, cerebrovascular, and cephalic vein, which suggested higher level of D-dimer was associated with more extensive and serious thrombosis.

Thrombosis can occur in the vessels of any part of the body (Table 5). Initially detection can occur as late as 31 days after disease onset (Table 1). However, thrombosis in the brain and abdomen may occur early; at 5 days after disease onset. It is possible that these forms of thrombosis are often very symptomatic, so they could be diagnosed earlier that other forms. Neurological symptoms such as cerebral infarction and hemiparesis, and severe gastrointestinal symptoms or even intestinal obstruction may be predominant severe and sometimes fulminant symptoms, as well as fever. Abdominal pain can be somewhat generalized in cases of spleen infarction [18, 30], which may not be detected via abdominal ultrasound but can be detected via abdominal enhanced CT [30]—as is consistent with the results of the present study. Chest pain was the most common symptom (Table 1), and accordingly pulmonary vessels were the most commonly involved sites (Table 5). Chest pain was often neglected because it was mild and/or intermittent. Patients with pulmonary vessel thrombosis are likely to experience back pain or abdominal pain (Table 1). As shown in Table 1, up to 35% (15/43) of patients were asymptomatic with regard to thrombosis, but D-dimer was an extremely important marker and early indicator in these patients. Intracardiac thrombosis can be the only type of thrombosis present in a patient, and it can be asymptomatic. It often occurs in the right heart chamber and close to the tricuspid valve [31]. Some large intracardiac thromboses have required surgical removal [17, 31]. Limb thrombosis often presents in association with typical features such as swelling or low temperature, but DVT and its associated symptoms can take a long time to resolve, even a year.

At least 22% of patients with pneumococcal NP exhibit indications of pulmonary infarction via microscopy [32]. In the present study 19/43 (44%) patients had NP. Among them, 16/19 (84.2%) had accompanying pulmonary vessel thrombosis, mainly PA thrombosis, which again suggested that NP was associated with pulmonary infarction due to PA thrombosis.

The mechanism underlying thrombosis due to MPP remains unknown. One suggested possible mechanism is that antiphospholipid antibodies induced by MP infection result in a transient hypercoagulable state, because positive ANA and aCL-IgM were found in more than 50% of patients with MP infection, especially MP-associated thrombosis [30, 33], which is consistent with the current study. Another direct invasion mechanism has been proposed in patients with stroke because MP-DNA was detected in cerebrospinal fluid [34]. MP bloodstream infection is rare and was detected via PCR in Case 1 in the current study, suggesting that MP may directly damage vascular endothelial cells, perhaps just as it damages airway epithelial cells. Some patients only had arterial involvement, such as pulmonary, intracranial, and/or abdominal artery thrombosis. PA thrombosis occurred on the same side as the pulmonary consolidation in 10 patients in the present study, and local arteritis was detected in Case 7. Furthermore, spleen infarction alone [30] and common carotid arteritis [35] have also been reported. The inflammation marker CRP may contribute to persistent obstruction of proximal PA by promoting vascular remodeling, endothelial dysfunction, and in situ thrombosis [36]. Therefore, we speculated that the isolated artery thrombosis was in situ thrombosis, which may explain the mild symptoms.

In the current study PLT counts gradually increased over time in most patients during the recovery period, and typical characteristics included an excessive immune response and a hypercoagulable stat. However, PLT are critical for haemostasis, thrombosis, pulmonary immune defenses, and inflammatory responses [37, 38]. The contribution of the lungs to PLT biogenesis is also substantial, accounting for approximately 50% of total PLT production [39]. Therefore, we speculated that PLT overactivation may play an important role in SMPP-associated thrombosis. PLT-fibrin complexes formed at the sites of vessel injury in the acute stage. With increased consumption of PLT, immature PLT from bone marrow and lungs was released into the peripheral circulation, therefore PLT increased during the recovery period. To date no studies investigating in situ thrombosis and dynamic PLT in MPP have been reported, and such studies may guide future mechanism research.

It has been reported that posterior cerebral artery occlusion after MP infection was associated with genetic defect of *MTHFR* C677T in a patient [20]. In the present study WES was performed in three patients with family histories of stroke, and it identified a heterozygous variant in *MTHFR* or in *DSG2* in each patient. Both *MTHFR*-associated thrombophilia and *DSG2*-associated arrhythmogenic right ventricular dysplasia or cardiomyopathy were autosomal dominant inherited disorders. Although we are not sure whether the above mutations are pathogenic, there are probably susceptibility genes such as *MTHFR* in these patients.

Unlike in adults, pediatric PE and intracardiac thrombosis often appears clinically silent. On retrospective review of children with an eventual diagnosis of PE, however, symptoms or signs were often present but may have been missed [40]. Therefore, PE even intracardiac thrombosis may be underestimated especially in SMPP with respiratory failure. The American Society of Hematology guideline panel developed detailed guidelines for the treatment of pediatric venous thromboembolism in 2018 [16]. The guidelines recommend or suggest (1) using anticoagulation in symptomatic DVT or PE; (2) either using anticoagulation or no anticoagulation in asymptomatic DVT or PE; (3) anticoagulation alone should be used in submassive PE; (4) using thrombolysis followed by anticoagulation in PE with hemodynamic compromise; (5) against using thrombectomy followed by anticoagulation; rather, anticoagulation alone should be used in symptomatic DVT or PE; (6) against using thrombolysis or surgical thrombectomy followed by standard anticoagulation; rather, anticoagulation alone should be used in right atrial thrombosis [16].

The absorption of thrombosis can be slow, therefore due attention should be paid to chronic thromboembolic PAH. In the current study transient PAH was only evident in Case 9, and that patient was the only one who received thrombolysis therapy. In some of the patients occlusion was detected in the airway, cerebrovascular vein, and PV, but all these patients were ultimately almost asymptomatic. In Case 10 there was right anterior cerebral artery occlusion, which suggested that brain function was probably transferred from damaged areas to non-damaged areas. There was intracranial venous occlusion in Case 6, but 3D black-blood 3T-MRI of the brain revealed lateral branch circulation. Therefore, to some extent collateral circulation may have occurred in these patients with vessel occlusion.

The present study had several potential limitations. WES was only performed in only three families. The reason for the small number of blood samples is that the MP-PCR-negative rate is very high, in MPP, thus clinicians do not often request blood samples for MP-PCR detection.

## Conclusions

SMPP with pulmonary consolidation (> 2/3 lobe) and a high level of inflammatory markers (CRP > 97.5 mg/L and LDH > 735.1 IU/L) were risk factors that were strongly associated with thrombosis. Symptoms associated with thrombosis such as chest pain may be subtle, and some patients may be asymptomatic. Therefore, keeping a high index of suspicion for thrombosis in children with SMPP is critical. Contrast-enhanced lung CT, echocardiography, and blood vessel ultrasonography should be routinely performed in SMPP patients with a high level of D-dimer, specifically > 11.1 mg/L (even > 5.0 mg/L).

## Data Availability

The datasets generated during and/or analysed during the current study are available from the corresponding author on reasonable request.

## Abbreviations

aCL: anticardiolipin
AO: airway obliterans
CAP: community-acquired pneumonia
CRP: C reactive protein
DVT: deep venous thrombosis
LA: lupus anticoagulant
LDH: lactate dehydrogenase
LMWH: low molecular weight heparin
MP: mycoplasma pneumoniae
MPP: mycoplasma pneumoniae pneumonia
NP: necrotizing pneumonia
PA: pulmonary artery
PE: pulmonary embolism
PLT: platelet
PV: pulmonary vein
SMPP: severe MPP
WES: whole exome sequencing
